# Safety of gadolinium based contrast agents in magnetic resonance imaging-guided radiotherapy – An investigation of chelate stability using relaxometry

**DOI:** 10.1016/j.phro.2022.02.015

**Published:** 2022-02-26

**Authors:** Faisal Mahmood, Ulla Gro Nielsen, Christian Brandt Jørgensen, Carsten Brink, Henrik S. Thomsen, Rasmus Hvass Hansen

**Affiliations:** a Laboratory of Radiation Physics, Department of Oncology, Odense University Hospital, Kloevervaenget 19, Indgang 85, Pavillonen, Stuen, 5000 Odense C, Denmark; b Department of Clinical Research, University of Southern Denmark, Winsløwparken 19, 3. Sal, Odense C 5000, Denmark; c Department of Physics, Chemistry and Pharmacy, University of Southern Denmark, Campusvej 55, 5230 Odense M, Denmark; d Faculty of Medical and Health Sciences, University of Copenhagen, Borgmester Ib Juuls vej 17, 2730 Herlev, Denmark; e Department of Radiology, Herlev and Gentofte Hospital, Borgmester Ib Juuls vej 17, 2730 Herlev, Denmark; f Department of Oncology, Rigshospitalet, Blegdamsvej 9, 2100 København Ø, Denmark

**Keywords:** Gadolinium based contrast agent, Magnetic resonance imaging, Radiotherapy, Safety, Toxicity, NMR relaxometry

## Abstract

**Background and purpose:**

With the introduction of hybrid magnetic resonance linacs (MR-linac), improved imaging has enabled daily treatment adaptation. However, the use of gadolinium based contrast agents (GBCAs) is desired to further improve MR image contrast. GBCAs are in the form of a non-toxic metalorganic gadolinium complex, but toxic un-chelated aqueous gadolinium(III), Gd^3+^(aq), can be released in patients if the organic ligand is degraded by the radiation. In this study, T_1_ relaxation measurements were performed to study the effect of radiation on three GBCAs.

**Materials and methods:**

GBCAs, gadoteric acid, gadobutrol and gadoxectic acid were investigated in a concentration range of 10–100 mM. Measurements were performed on a 500 MHz nuclear MR (NMR) spectrometer with a high-resolution inversion recovery sequence to determine T_1_. Samples were irradiated with 7 MV photons on an MR-linac to a total dose of 100 Gy. The lower detection limit of Gd^3+^(aq) was established by estimating the overall measurement uncertainty and comparing to corresponding changes in R_1_ when replacing chelated Gd^3+^ with gadolinium nitrate at predefined percentages.

**Results:**

The overall measurement uncertainty was estimated to ±0.0053 ms^−1^, corresponding to Gd^3+^(aq) detection levels 1%–1.5% or 1–4.5 micro molar at clinical GBCA dosage. No detectable differences in R_1_ were observed between irradiated and non-irradiated samples for any GBCA.

**Conclusions:**

This study did not find any measurable degradation of GBCAs due to irradiation with high-energy X-rays, however, in-vivo investigations are needed to provide the clinical basis for safe use of contrast agents in a radiotherapy workflow.

## Highlights

• No change in Gd-chelate relaxation rate after irradiation with high-energy X-rays.
• Complete breakage of Gd-chelate by high-energy X-rays not detected.
• In-vitro, complete breakage of Gd-chelate is below clinically relevant level.

## 1 Introduction

In recent years, multi-modality imaging has become an essential part of radiotherapy (RT) planning. Especially, MRI has gained a lot of popularity during the last decade mainly due to its superior soft-tissue contrast in comparison to CT and PET. Many RT departments have their own MR scanner, configured to accommodate treatment planning (so-called MR simulators) and it has become an integrated part of the planning workflow [1]. With the recent clinical introduction of hybrid MRI linear accelerator systems (MR-linacs), MRI has now moved into the treatment room for direct image guidance to provide better anatomical alignment of the patient, and facilitating daily treatment plan adaptation [2], [3].

Exogenous gadolinium based contrast agents (GBCAs) can be used to measure perfusion and to improve contrast between normal tissue and tumor. Gadolinium as bulk metal is ferromagnetic, but with sufficient atomic separation acts as a paramagnetic substance and shortens both T_1_ and T_2_ relaxation times [4]. For most anatomical imaging T_1_ shortening dominates, leading to signal enhancement in T_1_-weighted images. For example, liver lesions are likely indications for MRI-linac treatment and may stand out more clearly after intravenous administration of gadoxectic acid [5]. This implies higher treatment accuracy in some patients with contrast-enhanced images for treatment adaptation in the MR-linac workflow, as the subsequent dose delivery can rely directly on a visualization of the target rather than on surrogate structures.

As the time from imaging to end of dose delivery in the MR-Linac workflow is about 30 min [6], significantly shorter than the normal elimination half-life (1.6 h) of GBCAs in patients with normal renal function [7], irradiation of the contrast agent is unavoidable. Moreover, in some MR-linac workflows cinematic MRI during irradiation is used for verification of correct target and organ at risk positions and for automatic interruption of irradiation (beam gating) if the target moves outside the planned position [8].

Clinical GBCAs are in the form of a non-toxic metalorganic gadolinium complex, where the ligand is a polydentate, strong complexing agent. However, un-chelated gadolinium (Gd^3+^(aq)) can be released if large concentrations of other cations are presents or the organic ligand is degraded [9]. Degradation in the form of complete breakage or conformational changes of the chelate might occur during delivery of radiotherapy with the MR-linac, since X-rays used for treatment carry much higher mean energy compared to the bond dissociation energy of chemical compounds. The consequence of this can be deposition of Gd^3+^ in the body, which is known to have serious adverse health effects [10].

Nuclear spin relaxation is caused by the magnetic dipole–dipole interaction between the unpaired f-electrons in the gadolinium(III) ion (Gd^3+^, S = 7/2) and water molecules in the first coordination sphere of the GBCA complex, i.e. directly coordinated to Gd^3+^. By fast chemical exchange of these loosely bound water molecules and bulk water solvent the relaxation effect is further mediated. In addition, a secondary effect from water molecules diffusing around the complex within a second coordination sphere, i.e. in close spatial proximity but not directly bonded, is also present [11]. This results in a GBCA concentration dependent relaxation of the water signal. In particular, un-chelated Gd^3+^(aq) and complex bound Gd^3+^ are expected to have different coordination and relaxation properties.

A recent investigation, published during the submission of the current manuscript, used mass spectroscopy to demonstrate that the ionic chelate, gadobenate dimeglumine, and the macro cyclic chelate, gadobutrol, remain stable following irradiation [12]. However, the authors did not report the sensitivity of their method, making it difficult to evaluate its clinical relevance. Moreover, a time delay of up to seven days between irradiation and measurement was employed, which could potentially hide transient molecular changes. In one other report, from 2009, MR relaxometry was used to investigate the effect of radiation on an ionic linear and a non-ionic macrocyclic Gd-chelate [13]. The authors described a small effect of irradiation, however based on measurements using a clinical MRI system where precision is low compared to NMR spectrometer relaxometry where field homogeneity is higher and temperature is stable. In addition, the investigated GBCAs in that study have little relevance for most MR-linac indications, including the liver.

In this study, T_1_ relaxation measurements were performed on an NMR spectrometer to study the effect of radiation on GBCAs. Three commercially available GBCAs, currently used in radiotherapy planning of liver, CNS, and whole-body, were probed with T_1_ relaxation rates as a function of gadolinium concentration and radiation exposure, to assess possible irradiation induced dissociation of the gadolinium chelate. The sensitivity of the method was assessed and discussed in the context of existing toxicity data.

## 2 Materials and methods

### 2.1 Contrast agent sample preparation

The commercially available contrast agents Dotarem (0.5 mol/L gadoteric acid, ionic macrocyclic Gd-chelate, Guerbet), Gadovist (0.25 mol/L gadobutrol, nonionic macrocyclic Gd-chelate, Bayer) and Primovist (1 mol/L gadoxectic acid, ionic linear Gd-chelate, Bayer) were investigated as well as a 0.5 M Gd^3+^ solution prepared from gadolinium nitrate hexahydrate (99.9 % Sigma-Aldrich). Samples for liquid state ^1^H NMR were prepared directly in a 5 mm NMR tube (Wilmad) with a total volume of 500 μL of which 50 μL were D_2_O (99.90% & D Euriso-top) to facilitate the use of ^2^H lock. Samples with concentrations of 10, 20, 30, 40, 50, and 100 mM were prepared of the three commercial contrast agents and Gd^3+^. The required amount was extracted directly from the vial; 50 μL D_2_O and an appropriate amount of MiliQ® water was added. Samples were thoroughly mixed before measurement. All series were prepared in duplicates. In addition, 20 mM mixtures of a contrast agent and Gd^3+^ with 0, 5, 10, 50, and 100 mol% Gd^3+^ were prepared by mixing gadolinium nitrate (Gd(NO_3_)_3_) and the contrast agent in an appropriate amount to probe the detection limit. Irradiation was performed on 10, 20, 30, 40, 50, and 100 mM samples of gadoteric acid (in duplicate), and only on 20 mM samples of gadobutrol and gadoxectic acid (in triplicates), to limit the total number of measurements.

### 2.2 Sample irradiation

A specially crafted phantom was used to enable irradiation of seven NMR vials (samples) simultaneously. The radiation was delivered with a clinical MR-linac system (Unity, Elekta Instrument AB, Stockholm, Sweden) using 7 MV photons and a total dose of 100 Gy. A robust treatment plan was made based on a CT of the phantom using the clinical treatment planning system (Monaco, Elekta Instrument AB Stockholm, Sweden) to secure correct dose deposition to the whole sample. The phantom was setup in the planned position using the MV on board imaging system.

### 2.3 NMR relaxometry

^1^H NMR measurements were performed on a JEOL ECZ 500R 500 MHz spectrometer (Japan) equipped with a 5 mm Royal liquid state NMR probe. T_1_ was determined using an inversion recovery sequence (180-τ-90-acquisition) with 46 τ values ranging from 50 μs to 200 ms, a 90° (180°) pulse of 6.1 µs (12.2 µs), a relaxation delay of 10 s to ensure full relaxation, and 4 scans per spectrum . Each sample was measured at three time points, corresponding to before irradiation, 2 and 48 h after irradiation. We note that it was not possible to obtain spectra for gadoxectic acid and gadoteric acid with concentrations above 50 mM and 100 mM, respectively, as these samples could not be locked due to strong paramagnetic effects.

### 2.4 R_1_ estimation

The signal expression of an inversion recovery experiment is given as

$$s\left(TI\right)=s_{0}\left(1-2e^{-\frac{\mathrm{TI}}{T_{1}}}\right)$$

From this, it follows that $s\left(\infty \right)=s_{0}$, which was approximated by measuring the signal at $\mathrm{TI}\;>\;5T_{1}$. Rewriting the above equation using $Y\left(TI\right)\equiv ln(\frac{s_{0}-s\left(TI\right)}{s_{0}}$) gives

$$Y\left(TI\right)=-\frac{1}{T_{1}}TI+\ln\left(2\right)$$

from which $-\frac{1}{T_{1}}$ was estimated as the slope of the least square fitted line from paired values ($\mathrm{TI},Y\left(TI\right)$) [14] using Matlab R2019b Update 5 (The Mathworks, Inc., Natick, MA, USA). The inverse of the $T_{1}$ relaxation time, defined as the relaxation rate $\left(R_{1}\equiv \frac{1}{T_{1}}\right)$, was used as a convenient metric as it changes linearly with the concentration of GBCAs at low concentrations relative to the solvent concentration. The coefficient of change (slope) is known as the relaxivity.

### 2.5 Measurement uncertainty and Gd^3+^(aq) detectability

The overall measurement uncertainty was estimated using a control sample set, which consisted of six NMR tubes (*N* = 6) with gadoteric acid at the same concentrations as used for the irradiated samples. They were handled as the irradiated samples, except for the setup for dose delivery in the MR-linac treatment room.

NMR measurements of the control samples were performed at the same time points as the samples receiving radiation, i.e. before irradiation, 2 h after irradiation, and repeated 48 h after irradiation. For each set of repeated control sample measurements (labelled *i*), the relaxation rate (R_1_) uncertainty was calculated as the standard deviation (SD_i_) of the measured values. All these uncertainty evaluations were combined using root mean square to a single estimate of the overall measurement uncertainty. Finally, the value was multiplied by 1.96 to estimate the 95% confidence interval of the measurement uncertainty: $1.96\sqrt{\frac{1}{N}\sum_{i}^{N}{\text{SD}}_{i}^{2}}$. The minimum detectable amount of Gd^3+^(aq) was determined from a series of samples with a constant total Gd concentration (20 mM) with different relative concentrations of Gd^3+^(aq) and contrast agent (corresponding to 0, 5, 10, 50, and 100% Gd^3+^) prepared as described above. The minimum detectable concentration was defined as the concentration leading to a change in relaxation rate (R_1_) larger than the 95% confidence interval of the measurement uncertainty described above.

## 3 Results

### 3.1 Measurement uncertainty

The overall measurement uncertainty of R_1_, based on triplicate NMR measurements of gadoteric acid samples at each of the six different concentration levels (18 measurements in total), was estimated to ±0.0053 ms^−1^ at 95% confidence level. Thus, changes in R_1_ less than this value cannot reliably be detected in the current study.

### 3.2 Relaxation rates (R_1_)

**Fig. 1:**
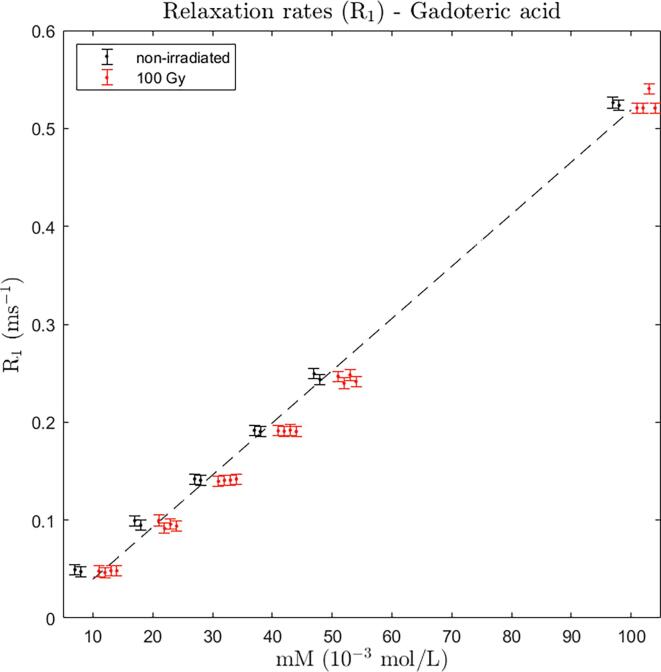
Relaxation rates (R_1_) for gadoteric acid at concentrations 10–100 mM. For each concentration, two (black) data points for the pre-irradiation condition (non-irradiated) and four (red) data points (two at 2 h and two at 48 h after irradiation) for the post-irradiation condition (100 Gy) are shown. Data points belonging to same concentration are slightly moved apart on the x-axis for better visualization. The error bars indicate the estimated 95% CI of R_1_ in this study (±0.0053 ms^−1^). Dashed line shows a linear fit based on pre-irradiation data points. (For interpretation of the references to colour in this figure legend, the reader is referred to the web version of this article.)

*Gadoteric acid:* Two independent sets of samples consisting of six different concentration each (10, 20, 30, 40, 50, and 100 mM) were measured. A linear relation between R_1_ relaxation rates and the concentrations was observed (Fig. 1). It is seen that the difference in pre- and post-irradiation (2 and 48 h) relaxation rate was within the measurement uncertainty which is indicated as 95% CI error bars (±0.0053 ms^−1^). The observed post-irradiation relaxation rates were reduced in mean by 0.00076 ms^−1^ which is about 14% of the CI (*P* = 0.22, paired two-tailed *t*-test).

**Fig. 2:**
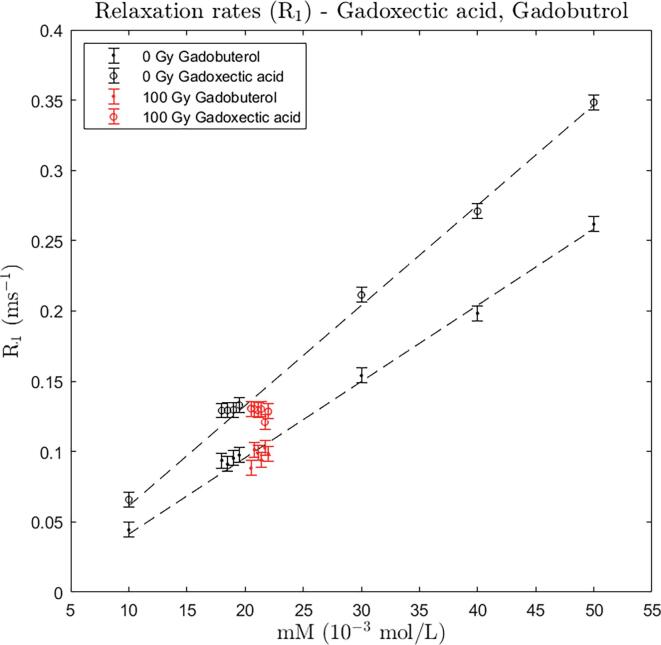
Relaxation rates (R_1_) for gadobutrol and gadoxectic acid at concentrations 10–50 mM for control samples (one sample per concentration). For 20 mM, three (black) data points for the pre-irradiation condition and six (red) data points (at 2 h and 48 h after irradiation) for the post-irradiation condition are shown. Data points belonging to same concentration are slightly moved apart on the x-axis for better visualization. The error bars indicate the estimated 95% CI of R_1_ in this study(±0.0053 ms^−1^). Dashed line shows a linear fit based on control data points. (For interpretation of the references to colour in this figure legend, the reader is referred to the web version of this article.)

*Gadoxectic acid and gadobutrol:* The relaxation rates of gadoxectic acid and gadobutrol control samples (0 Gy), respectively, at concentrations 10, 20, 30, 40, and 50 mM show a linear relationship, with the gadoxectic acid having the highest relaxivity (Fig. 2). For both contrast agents triplicate of samples at 20 mM were measured before irradiation and twice after irradiation with 100 Gy (at 2 h and 24 h). It is seen that the difference between pre- and post-irradiation samples were within the measurement uncertainty which is indicated as 95% CI error bars (±0.0053 ms^−1^). The observed post-irradiation relaxation rates were reduced by 0.0026 ms^−1^ for gadoxectic acid and increased by 0.0049 ms^−1^ for gadobutrol, corresponding to about 49% and 92% of the CIs, respectively.

### 3.3 Gd^3+^(aq) detectability

**Fig. 3:**
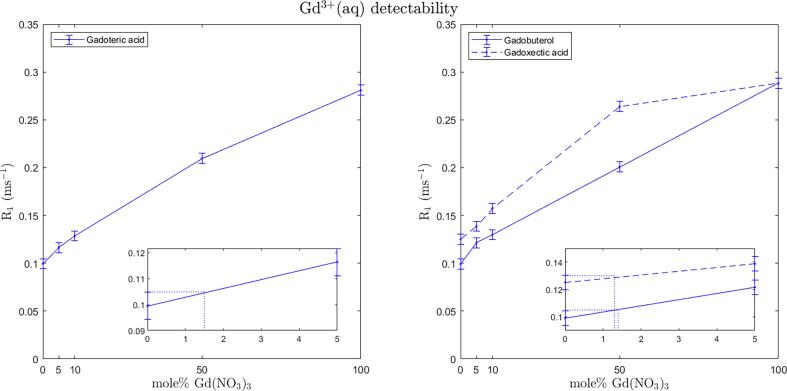
Gd^3+^(aq) detectability in gadoteric acid (left), gadobutrol and gadoxectic acid (right). Total gadolinium concentration (chelated and un-chelated) of samples is 20 mM. Error bars indicate the overall measurement uncertainty of ±0.0053 ms^−1^ corresponding to 95% CI. The 0–5 mol% of gadolinium nitrate range is enlarged in separate plots in the lower right corners of the overall plots to show the detection thresholds of Gd^3+^(aq) in the three GBCAs.

R_1_ increases with increasing percentages of the Gd(NO_3_)_3_ since Gd^3+^ (aq) has a presumably smaller coordination sphere leading to an, on average, stronger dipole interaction by the fluctuating magnetic field from the seven unpaired electrons of Gd with the solvent. In general, the linear relation between relaxation and concentration holds for low concentrations of the contrast agent and the ranges of linearity are different for GBCAs and Gd^3+^(aq). The R_1_ relaxation of the mixtures in this case deviated from linearity as the concentration of Gd^3+^(aq) increased (Fig. 3), indicating that the range of linearity for the salt was exceeded. The error bars representing the overall measurement uncertainty at 95% CI level, indicated that the lower limit of Gd^3+^(aq) detectability was about 1.5% in gadoteric acid solution (Fig. 3), or about 0.3 mM Gd^3+^(aq) in a 20 mM solution. For gadobutrol and gadoxectic acid the detection levels were found to be about 1% and 1.5% (0.2 mM and 0.3 mM out of 20 mM), respectively (Fig. 3).

## 4 Discussion

MRI is becoming an essential modality in radiotherapy planning. With the recent arrival of in-room MRI-guided radiotherapy systems, patients with soft tissue lesions can potentially be treated with higher accuracy and precision. For some indications, GBCAs are required for increased image conspicuity to outline the target clearly for plan adaption before dose delivery. Uncertainty about the chemical stability of GBCA after irradiation motivated the current investigation. This study, using T_1_ relaxometry, did not find any measurable degradation of any of the investigated GBCAs due to irradiation with high-energy X-rays.

This study supports the findings of Wang et al. who published shortly after submission of the current manuscript. In their study mass spectroscopy was used to detect Gd-chelate breakage when up to 30 Gy was deposited to GBCA samples [12]. In the current investigation, we used a radiation dose of 100 Gy and in addition established a detection limit, which was not reported by Wang et al. This allows us to discuss the clinical relevance of our results.

NMR relaxometry depends on the hydration number of the chemical complex and as such is an indirect method for measuring chemical changes. In the current study, a complete breakage of the chelate leading to a release of Gd^3+^(aq) was modelled by replacing predefined quantities of the non-irradiated GBCA with a gadolinium salt (gadolinium nitrate). Based on the change in relaxation rates of the mixture the detection level of the method was found to be in the order of 1–1.5% of the contrast agent concentration. The literature on clinical toxicity levels is sparse, but suggests an LD50 levels of about 0.1–0.3 mmol/kg in mice [15], which is equivalent to 0.1–0.3 mM Gd^3+^(aq) in solutions. The standard human dose of the investigated GBCAs is in the range 0.1 to 0.3 mmol/kg. At this dosage, the results indicate that 1–1.5% of 0.1–0.3 mmol/kg, or equivalently, about 1 to 4.5 micro molar of Gd^3+^(aq) may be released in the patient, which is 1–1.5% of LD50. This is a conservative estimate, since the administered GBCA is distributed throughout the body, whereas radiation dose is delivered loco-regionally. The irradiated portion of the GBCA will eventually diffuse and flow to other body parts before clearing, thus further dilution of possible un-chelated molecules is expected. The current study used an overall estimation of the measurement uncertainty across GBCAs. Although this is deemed a reasonable assumption, ideally should be estimated individually to achieve GBCA-specific detection limit estimates.

The results in the current study do not indicate potential release of Gd^3+^(aq) due to high energy X-ray irradiation that might lead to clinical toxicity. However, some important caveats need to be mentioned: Local retention of Gd^3+^(aq) in tissues such as brain, CSF, bone, skin and liver could still induce morbidity at low concentrations [16], [17], and repeated use of GBCAs during the radiotherapy course could potentially accumulate in the patient [18], reaching total levels that may give late adverse reactions. On the other hand, no adverse effects have been found in case of accumulation of Gd in the brain or other tissues, in patients with normal kidney function (www.fda.gov).

One of the limitations of this study is that in-vitro samples of GBCAs were used to model the clinical situation. This may not be an ideal approach since in patients the GBCAs are irradiated in the intravascular or interstitial space, where a different chemical environment is present (several ions and metabolites such as N-acetylaspartate, Choline, Creatine and Myo-inositol). In theory, radiation-induced damage to the gadolinium chelate may be reverted more easily in a pure water environment than in-vivo, where Gd^3+^(aq) may precipitate as insoluble gadolinium phosphate leading to depositions [19]. Even under non-irradiated conditions, it has been shown that the stability of GBCAs in the human body may be reduced drastically, compared to their stability in standard solutions, especially in patients with impaired renal function [20]. In addition, the relaxation measurements could not be performed sooner than 2 h after irradiation, implying that possible rapid transient conformational changes of the Gd-chelate could not be detected. Such changes could under in-vivo condition interact with the local chemical environment and become permanent and internalized.

The results of this study may also be relevant for situations other than the in-room MR-linac workflow. In patients with normal renal function, the mean half-lives for distribution and clearance of commonly used GBCAs are about 12 min and 96 min, respectively [21]. This means that MRI contrast administered in the radiotherapy preparation phase, typically several days before start of treatment, reside in the patient at very small concentrations by the time of treatment. But, patients participating in clinical studies might be scanned with GBCAs within hours of the scheduled radiotherapy treatment [22], demanding some awareness, for example by designing the trial with a generous gap between scan and treatment, unless a short gap is critical for the investigation.

Follow-up studies are important to validate the results of the current study in an in-vivo design, which models the clinical situation more accurately. This is particularly important in order to investigate retention of Gd^3+^(aq) in the tissue, and the possible gadolinium accumulation effect, to imitate repeated use of GBCAs during a radiotherapy course. The latter may be especially relevant for linear Gd-chelates that show several fold higher tissue retention levels than macrocyclic Gd-chelates [23]. The accumulation effect should be taken into account since risk of long-term toxicity may potentially be higher when retained GBCAs are irradiated over many fractions. Investigation of the feasibility of using reduced GBCA doses is also needed, since standard protocols from radiology may not be optimal for on-line adaptation workflows in radiotherapy. An optimized protocol should recommend minimum GBCA dose while maintaining sufficient image conspicuity.

In conclusion, no change in relaxation rates was observed after deposition of sup-clinical radiation dose into solutions of three different commercially available GBCAs (both linear and macrocyclic). Thus, this study did not find any measurable degradation of the GBCAs due to irradiation with high-energy X-rays. The demonstrated lower limit of 1.5% to detect un-chelated gadolinium, infers that the investigated GBCAs, under in-vitro conditions, releases less than 4.5 micro molar of un-chelated gadolinium, which can be considered below critical level in a clinical situation. However, follow-up in-vivo studies are needed to draw clear conclusions for the safety in patients.

## Declaration of Competing Interest

The authors declare that they have no known competing financial interests or personal relationships that could have appeared to influence the work reported in this paper.
